# Supplementary material for: Synergistic Effects of DHA and Sucrose on Body Weight Gain in PUFA-Deficient Elovl2 -/- Mice
Source: Nutrients. 2019 Apr 15;11(4):852. doi: 10.3390/nu11040852 (PMC6520711; doi:10.3390/nu11040852)
Supplement: Supplementary file 1 [file nutrients-11-00852-s001.pdf]

**Supporting information**

**Table S1. Primer sequences**

| ***Genes*** | ***Primer*** | ***Sequence*** |
|---|---|---|
| GLUT4 | Forward | GCTTTGTGGCCTTCTTTGAG |
| | Reverse | CAGGAGGACGGCAAATAGAA |
| CPT1 | Forward | GCGCTCTTAGGACTACTTGCTAACC |
| | Reverse | ACTGGAGACCTGAGAGAGGAATGT |
| PPARgamma | Forward | GAAAGACAACGGACAAATCACC |
| | Reverse | GGGGGTGATATTTTGAACTTG |
| Elovl3 | Forward | GCCTCTCATCCTCTGGTCCT |
| | Reverse | TGCCATAAACTTCCACATCCT |
| CART | Forward | TGGATGATGCGTCCCATG |
| | Reverse | CGGAATGCGTTTACTCTTGAGC |
| NPY | Forward | CGCTCTGCGACACTACATCAA |
| | Reverse | GGGCTGGATCTCTTGCCAT |
| AgRP | Forward | GGTGCTAGATCCACAGAACCG |
| | Reverse | CCAAGCAGGACTCGTGCAG |
| POMC | Forward | CGAGGCCTTTCCCCTAGAGT |
| | Reverse | CCAGGACTTGCTCCAAGCC |
| leptin | Forward | GTGGTGGCTGGTGTCAGAT |
| | Reverse | TTGATGAGGTGACCAAGGTG |
| adiponectin | Forward | GGAGAGAAAGGAGATGCAGGT |
| | Reverse | CTTTCCTGCCAGGGGTTC |
| 18S | Forward | AGTCCCTGCCCTTTGTACACA |
| | Reverse | CGATCCGAGGGCCTCACTA |
| TFIIB | Forward | GTTCTGCTCCAACCTTTGCCT |
| | Reverse | TGTGTAGCTGCCATCTGCACTT |

**A**

| | Wt | KO |
|---|---|---|
| HS (control) | 87,02 ± 0,33 | 87,33 ± 0,24 |
| HS | 87,33 ± 0,38 | 87,56 ± 0,24 |
| HSDHA | 86,93 ± 0,14 | 87,37 ± 0,11 |

**B**

| | Wt | KO |
|---|---|---|
| HS (control) | 87,19 ± 0,27 | 87,55 ± 0,16 |
| HS/HSHF | 87,31 ± 0,27 | 87,30 ± 0,06 |
| HSDHA/HSHF | 87,33 ± 0,06 | 87,55 ± 0,10 |

**Table S2. Apparent food absorption efficiency**

Apparent mass absorption efficiency calculated as the ratio of the ingested mass minus the excreted mass (feces mass) to mass ingested at two time points: at the shifting point from DHA enriched or deficient diet to high fat diet (**A**) and after 2 weeks of high fat diet treatment (at week 6) (**B**). Values were normalized by arcsine transformation.

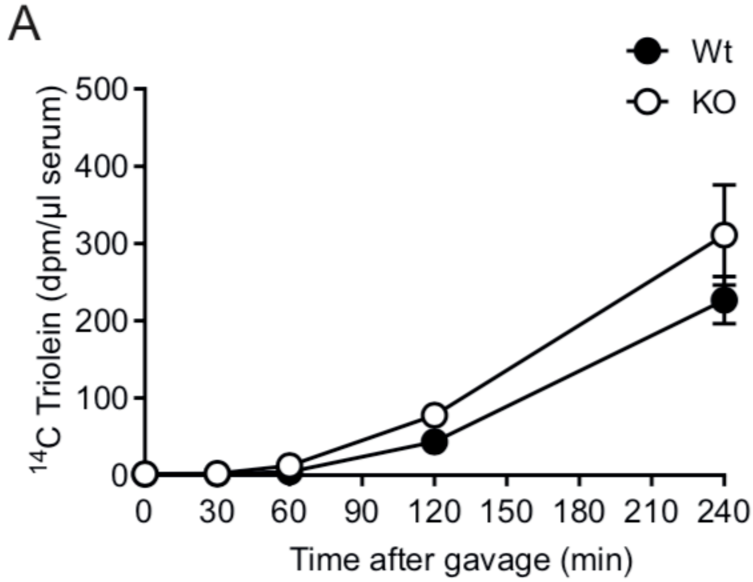


**Figure S1. Intestinal lipid uptake test**

18-20-week-old wild-type and *Elovl2-/-* mice, maintained on low-sucrose diet, were fasted overnight and then injected with tyloxapol (Lipase inhibitor) 15 minutes prior to gavage of olive oil traced with $^{14}C$-triolein. Blood was collected 30, 60, 120 and 240 minutes post gavage to measure radioactive tracer recovery. Results shown are means of 6 mice ± SEM.

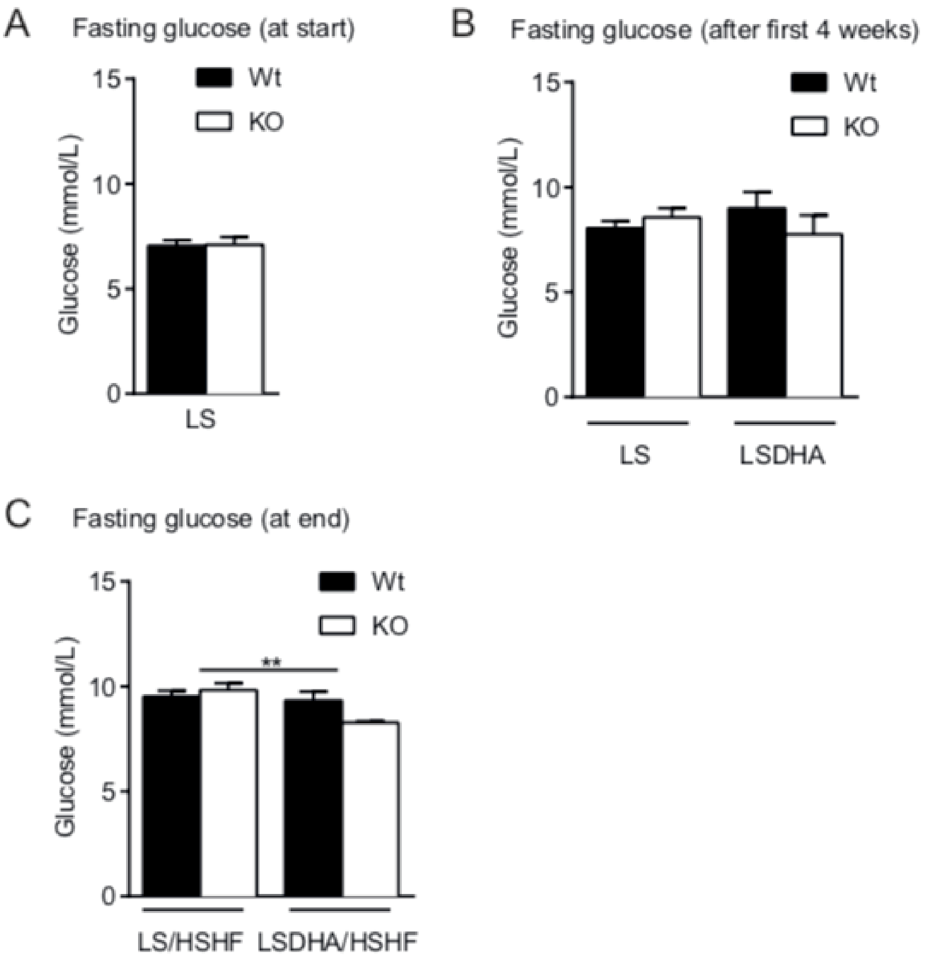


**Figure S2. Fasting glucose concentration in blood**.

Glucose levels in wild-type and *Elovl2-/-* mice were measured at the beginning of the experiment, after 4 weeks of pre-treatment with low-sucrose or low-sucrose DHA-enriched diet (LSDHA), and at the end of 4 weeks of high-sucrose, high-fat diet (HSHF) feeding. Animals were fasted for 6 hours prior to the analysis. Results shown are means of 4 mice ± SEM. Statistical significances are shown between groups **$P$<0.01.

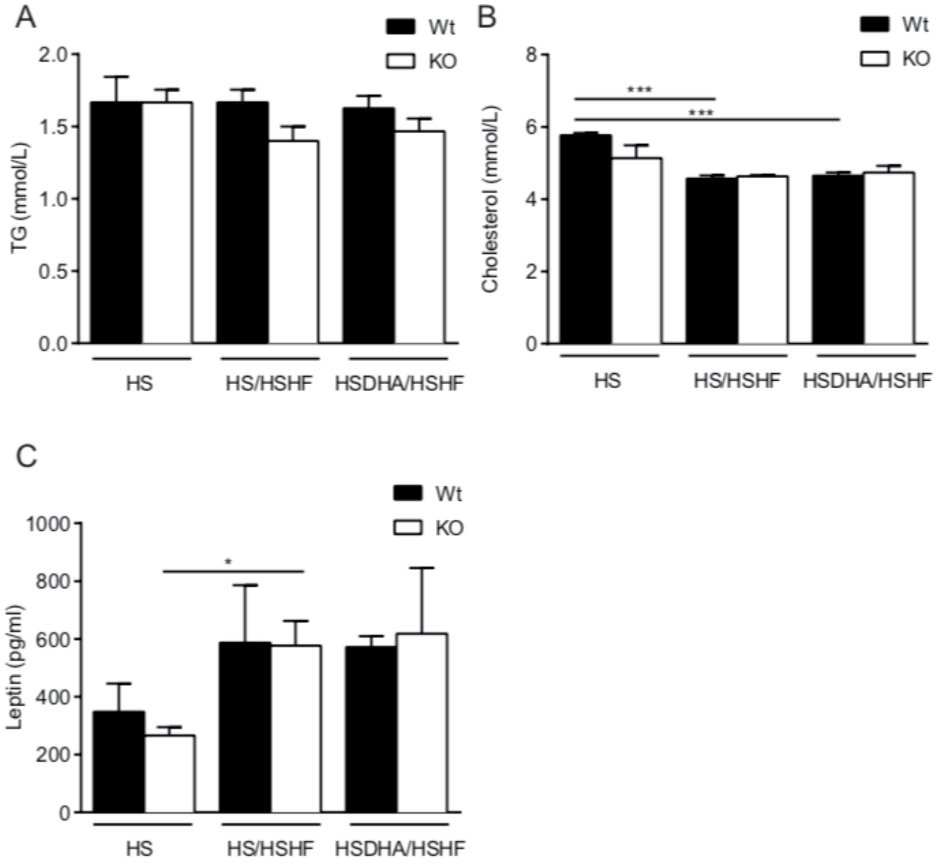


**Figure S3. Parameters of lipid metabolism in the blood.**

Triglyceride (TG), cholesterol and leptin levels were analyzed in blood and serum of wild-type and *Elovl2-/-* mice that were fed high-sucrose (HS) for 8 weeks and high-sucrose or high- sucrose DHA-enriched diet for 4 weeks followed by high-sucrose, high-fat diet (HS/HSHF and HSDHA/HSHF respectively) for 4 weeks. Serum samples for leptin and insulin measurements were collected at the end point of experiment; triglyceride and cholesterol levels were measured in after the GTT at 6 weeks of treatment (4 weeks of pre-treatment and 2 weeks of high-sucrose, high-fat diet (HSHF)). Results shown are means of 4 mice ± SEM. Statistical significances are shown between groups * $P<0.05$. ***$P<0.001$
